# Acupuncture Modulates Neurotransmitter‐Related Molecules in the Amygdala to Ameliorate Generalized Anxiety Disorder

**DOI:** 10.1002/cns.70847

**Published:** 2026-04-20

**Authors:** Zhao Sun, Yixiang Wang, Xiaona Kang, Binyan Ran, Qiong Wu, Luyu Huang, Jiaxin Li, Lina Kan, Haixia Long, Jiabin Liang, Wei Shen

**Affiliations:** ^1^ Traditional Chinese Medicine College, Hainan Medical University Haikou China; ^2^ Department of Acupuncture and Moxibustion First Affiliated Hospital of Hainan Medical University Haikou China; ^3^ Department of Traditional Chinese Medicine Preventive Healthcare Zhongshan Hospital of Traditional Chinese Medicine Zhong Shan China; ^4^ College of Information Science and Technology, Hainan Normal University Haikou China; ^5^ Chinese Medicine Guangdong Laboratory Zhuhai China; ^6^ The Second Affiliated Hospital Guangzhou University of Chinese Medicine Guangzhou China

**Keywords:** chronic unpredictable stress (CUS), electroacupuncture (EA), functional magnetic resonance imaging (fMRI), generalized anxiety disorder (GAD)

## Abstract

**Background:**

Neurotransmitter imbalance is a key mechanism contributing to the heightened negative emotions and anhedonia associated with anxiety disorders. However, whether acupuncture exerts its anxiolytic effects by modulating neurotransmitter imbalance remains unclear.

**Methods:**

Seventy generalized anxiety disorder (GAD) patients were recruited and randomly assigned to acupuncture or wait‐list groups. The Hamilton Anxiety Rating Scale (HAMA) and fMRI scans were used before and after treatment to assess anxiety levels and brain activity. In parallel, male Sprague–Dawley rats were divided into four groups: control, chronic unpredictable stress model (CUS), electroacupuncture (EA), and sham acupuncture (SA). The EA group received stimulation at specific acupuncture points over 21 days, while the CUS group experienced chronic unpredictable stress to induce anxiety. Behavioral assessments and molecular analyses, including qRT‐PCR, Western blotting, and immunofluorescence, measured neurotransmitter levels.

**Results:**

Clinically, the acupuncture group exhibited a significant reduction in HAMA scores compared to baseline (*p <* 0.001), unlike the wait‐list group. fMRI results indicated decreased ReHo in brain regions such as the amygdala, hippocampus, anterior and posterior cingulate cortices, putamen, and precuneus following acupuncture (GRF‐corrected *p*‐cluster < 0.05). These reductions showed overlap in the anterior and posterior cingulate cortices between both groups. A positive correlation was found between reduced ReHo in the amygdala and the decrease in HAMA scores (rs = 0.390, *p =* 0.023), while a negative correlation was observed between reduced ReHo in the hippocampus and illness duration (rs = −0.385, *p =* 0.025). In the animal model, EA improved body weight and reduced anxiety‐like behaviors (*p <* 0.05). EA increased IGF‐1 expression in the mPFC and amygdala, and decreased NR2B, GluR2 in the amygdala (*p <* 0.05).

**Conclusion:**

Acupuncture shows potential in treating anxiety disorders by modulating IGF‐1 and NR2B expression, thus restoring neurotransmitter balance in the mPFC‐amygdala pathway. In both animal models and clinical settings, acupuncture effectively reduced anxiety symptoms and induced positive changes in brain regions such as the amygdala and hippocampus. These findings provide preliminary evidence for using acupuncture in the treatment of GAD.

**Clinical Trial Number:**

MR‐46‐23‐043956.

## Graphical abstract

Acupuncture alleviates anxiety‐like behaviors by modulating functional brain regions and neurotransmitter‐related gene expression. In both human and animal models, this study confirms that acupuncture regulates amygdala activity, offering potential for anxiety management.

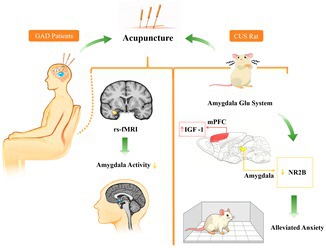

**Abbreviations**
ACC: Anterior Cingulate Cortex
ALFF: Amplitude of Low‐Frequency Fluctuations
BDI‐II: Beck Depression Inventory‐II
BSA: Bovine Serum Albumin
CE: Closed Arm Entries
CT: Closed Arm Time
CUS: Chronic Unpredictable Stress
DAPI: 4′,6‐Diamidino‐2‐Phenylindole
DARTEL: Diffeomorphic Anatomical Registration using Exponentiated Lie Algebra
EA: Electroacupuncture
EPM: Elevated Plus Maze
fMRI: Functional Magnetic Resonance Imaging
FOV: Field of View
GAD: Generalized Anxiety Disorder
Glu: Glutamate
GM: Gray Matter
HAMA: Hamilton Anxiety Rating Scale
IGF‐1: Insulin‐like Growth Factor 1
MNI: Montreal Neurological Institute
mPFC: Medial Prefrontal Cortex
MPRAGE: Magnetization‐Prepared Rapid Gradient Echo
MRI: Magnetic Resonance Imaging
mRNA: Messenger Ribonucleic Acid
NC: Normal Control
NIFTI: Neuroimaging Informatics Technology Initiative
NR2B: N‐Methyl‐D‐Aspartate Receptor Subunit 2B
OE: Open Arm Entries
OFT: Open Field Test
OT: Open Arm Time
PCC: Posterior Cingulate Cortex
PCR: Polymerase Chain Reaction
qRT‐PCR: Quantitative Real‐Time Polymerase Chain Reaction
ReHo: Regional Homogeneity
rs‐fMRI: Resting‐State Functional Magnetic Resonance Imaging
SA: Sham Acupuncture
sFC: Static Functional Connectivity
SPM: Statistical Parametric Mapping
TE: Echo Time
TR: Repetition Time
WM: White Matter

## 1 Introduction

Anxiety disorders are clinically characterized by heightened negative emotions and anhedonia, reflecting chronic and pathological emotional distress [1]. Research has shown that these disorders are associated with imbalances in neurotransmitters within specific brain regions [2]. Long‐term use of mood stabilizers like lithium and valproate has been observed to reduce synaptic AMPA receptor subunits, GluR1/2, in cortical neurons [3]. Bernardo et al. [4] found that enhancing GABAA receptor activity decreased immobility time in the forced swim test and improved spatial working memory impaired by stress. Electrophysiological studies indicate that IGF‐1 inhibits excitatory synaptic transmission in the sub‐limbic pyramidal neurons of rats, facilitating the extinction of fear memories [5].

Neurotransmitter imbalances within brain regions result in dysregulated signal transduction, inflammation, increased oxidative stress, mitochondrial dysfunction, and neuronal death. Accumulating evidence suggests that neurotransmitters play a central role in both the onset and persistence of anxiety disorders. Chronic stress‐induced overactivation of Glu and its receptors leads to neuron depolarization, with Glu NMDA receptor channel opening causing Ca2^+^ influx, subsequently damaging cells. Studies have shown a positive correlation between the intensity of glutamatergic signaling and affective symptoms [6, 7]. Electrophysiological experiments suggest that acute stress enhances synaptic transmission from the prefrontal cortex to the amygdala via excitatory circuits, temporarily increasing anxiety‐like behavior [8]. Inhibiting the expression of excitatory neurotransmitters can alleviate anxiety, underscoring the importance of targeting neurotransmitter imbalances as a therapeutic strategy for anxiety disorders.

Neuroimaging studies have revealed key neural mechanisms underlying anxiety disorders. Resting‐state functional MRI (rs‐fMRI) has identified altered low‐frequency amplitude (ALFF) in anxiety‐related brain regions. For example, Chen et al. [9] found elevated ALFF values in the left thalamus and hippocampus of generalized anxiety disorder (GAD) patients, correlated with somatic symptom severity. Wang C et al. [10] reported reduced static functional connectivity (sFC) between the medial amygdala and executive control network in high‐trait anxiety individuals, alongside extensive amygdala dysfunction. Abnormal mPFC volume in GAD correlates with anxiety disorders, with mPFC exerting top‐down inhibition on amygdala‐mediated negative emotional processing [11, 12, 13]. These findings highlight that dysfunction in the amygdala and prefrontal cortex underpins excessive anxiety, worry, and autonomic dysregulation in anxiety disorders.

Acupuncture has proven effective in alleviating anxiety‐like behaviors. The amygdaloid complex, or amygdala, is a crucial component of the limbic system, instrumental in regulating emotions and motivation [14]. GABAergic dysregulation within the amygdala was associated with stress‐related neuropsychiatric disorders [15]. The prefrontal cortex receives external stimuli and channels this information to structures regulating emotion, fear, and stress responses [16], such as the amygdala and the dorsal raphe nucleus. Anxiety disorders have been shown to affect the structure of the prefrontal cortex and its functional connectivity with distal brain structures [17]. The amygdala and prefrontal cortex are key regions where acupuncture exerts its effects [18, 19].

Based on these findings [20, 21, 22, 23], the current research employs acupuncture technique for the treatment of GAD. Clinically, this study identified differences in Regional Homogeneity (ReHo) values in key brain regions before and after acupuncture treatment using resting‐state fMRI. The correlation between the differences in ReHo in key brain regions and behavioral scales provides insight into its therapeutic efficacy, and also provides evidence for subsequent basic research. Experimentally, a chronic unpredictable stress (CUS) rat anxiety model was established and randomly divided into a blank control group, model group, electroacupuncture group, and sham acupuncture group. After 3 weeks of treatment, behavioral impact was assessed using the open field and elevated maze tests. Neurotransmitter‐related changes were analyzed by examining mRNA and protein expression levels of NR2B, GluR1, GluR2, and IGF‐1 in the amygdala and IGF‐1 in the medial prefrontal cortex. These combined neuroimaging and biological approaches aimed to elucidate the central regulatory mechanism of the anxiolytic effects of acupuncture, with a particular focus on neurotransmitter imbalances and their role in the medial prefrontal cortex‐amygdala axis, providing new perspectives for clinical treatment.

## 2 Materials and Methods

### 2.1 Clinical Study Design

**FIGURE 1:**
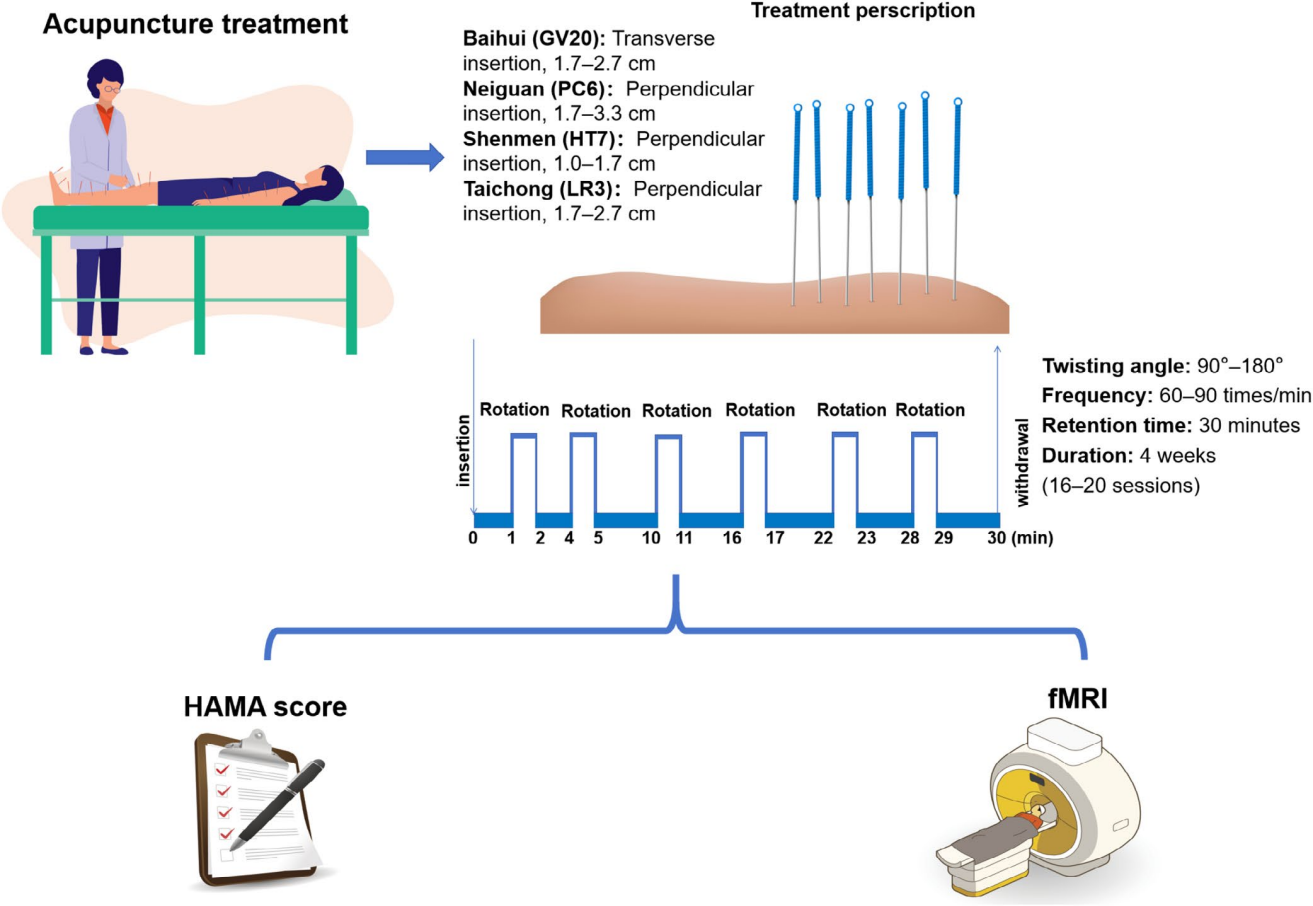
Clinical treatment schematic.

This study was a controlled trial designed to evaluate the effects of acupuncture therapy on patients with GAD by comparing outcomes before and after the intervention. A assessor‐blinded approach was implemented, wherein imaging analysts were blinded to the intervention to ensure objective data evaluation, while clinicians performing the acupuncture therapy were not blinded due to the nature of the treatment. The flowchart is shown in Figure 1.

### 2.2 Participants

The study recruited patients diagnosed with GAD who visited the clinic of the First Affiliated Hospital of Hainan Medical University between January 2023 and June 2024. A total of 70 eligible patients meeting predefined inclusion and exclusion criteria were enrolled and randomly allocated into either the acupuncture therapy group or the wait‐list control group using a computer‐generated randomization table. All participants underwent fMRI scanning before and after the observation period. The study protocol received ethics approval from the Institutional Review Board of the First Affiliated Hospital of Hainan Medical University (Approval No. [2023‐KYL‐068]), and written informed consent was obtained from all participants prior to enrollment.

### 2.3 Criteria

#### 2.3.1 Inclusion Criteria

1. Diagnosis of GAD per *Diagnostic and Statistical Manual of Mental Disorders, Fifth Edition (DSM‐5)* criteria [24].
2. Age between 20 and 60 years, with a disease course exceeding 6 months.
3. GAD‐7 score ≥ 10.
4. HAMA score ≥ 7.
5. Right‐handed with at least primary school education.
6. Normal or corrected visual acuity.

#### 2.3.2 Exclusion Criteria

1. Presence of other psychiatric disorders such as major depressive disorder or schizophrenia.
2. Pregnancy or lactation.
3. Initiation of new psychotropic medications or significant dosage adjustments during the trial period or prior acupuncture treatment in the past 3 months.
4. Severe suicide risk, cardiovascular diseases, or organ dysfunction.
5. MRI contraindications such as metallic implants or claustrophobia.

#### 2.3.3 Discontinuation and Dropout Criteria

1. Non‐compliance or failure to complete ≥ 80% of treatment sessions.
2. Use of treatments excluded by the study.
3. Self‐modification of treatment plans.
4. Excessive head motion during fMRI (movement > 1°).
5. Serious adverse events or complications requiring withdrawal.

### 2.4 Blinding

Due to the unique nature of acupuncture, a design was used. The acupuncturists were not blinded, but the personnel analyzing the imaging data were blinded to group assignments.

### 2.5 Acupuncture Intervention

Selected acupoints included Baihui (GV20), Neiguan (PC6), Shenmen (HT7), and Taichong (LR3). Acupuncture was performed according to the national standards (GB/T12346‐2006). Disposable sterile needles (0.25 mm × 25 mm and 0.25 mm × 40 mm) were used. The intervention involved bilateral stimulation of the above acupoints using uniform lifting, thrusting, and twisting techniques. Each session lasted 30 min, with treatments administered 4–5 times per week over 4 weeks (16–20 sessions). The wait‐list control group did not receive acupuncture treatment, and a minority of medicated patients maintained the original medication.

### 2.6 Behavioral Evaluation

The HAMA and the Beck Depression Inventory‐II (BDI‐II) were used for evaluation. The BDI‐II was administered prior to grouping to exclude patients with comorbid depression. In the acupuncture group, HAMA scores were assessed before and after treatment. In the wait‐list control group, HAMA scores were collected before the first scan and after the second scan.

### 2.7 MRI Outcome

#### 2.7.1 Scanning Protocol

MRI scans were conducted using a 3.0 T Signa EXCITE system (GE Healthcare, USA) in the Magnetic Resonance Room of the First Affiliated Hospital of Hainan Medical University. All patients with GAD underwent resting‐state fMRI scans within 3 days prior to acupuncture treatment and within 3 days following the completion of treatment. The wait‐list control group was scanned twice, with a 4‐week interval between the first and second scans. High‐resolution brain structural imaging was obtained using T1‐weighted three‐dimensional magnetization‐prepared rapid gradient echo (MPRAGE) sequence, with the following parameters: repetition time (TR) = 6.1 ms, echo time (TE) = 1.8 ms, flip angle = 9°, slice thickness = 1 mm, total volumes = 166 slices, field of view (FOV) = 256 × 256 mm, data matrix = 256 × 256 mm, voxel size = 1 mm. Resting‐state BOLD scanning duration was 6 min and 24 s. Brain functional imaging utilized a gradient echo EPI sequence with parameters as follows: TR = 3000 ms, TE = 35 ms, flip angle = 90°, slice thickness = 3.6 mm, interslice gap = 0 mm, total volumes = 41 slices.

#### 2.7.2 Data Processing

fMRI data processing and statistical analysis were performed using DPABI 7.0 and SPM12 on MATLAB R2022b, with the final results visualized in image format. Raw MRI data were converted from DICOM to NIFTI format, discarding the first 8 time points while retaining the remaining 120 for analysis. Slice timing correction and head motion correction were applied, excluding data with mean head motion exceeding ±1 mm or rotation greater than ±1°. Functional images were registered to each subject's T1 structural image, and gray matter (GM), white matter (WM), and cerebrospinal fluid (CSF) were segmented for transformation to standard space. The DARTEL method was used for estimating Friston 24 head motion parameters, with spatial normalization to the Montreal Neurological Institute (MNI) space. Regional Homogeneity (ReHo) values were calculated based on the similarity in time series between each voxel and its adjacent voxels. A band‐pass filter (0.01–0.08 Hz) removed linear drift. Statistical analysis was based on core brain regions of the limbic system as a mask, with a significance threshold of GRF (voxel *p* < 0.001, cluster *p* < 0.05).

## 3 Animal Experiments

### 3.1 Animals

Healthy male Sprague–Dawley rats, weighing approximately 160–180 g, were purchased from Changsha Tianqin Biotechnology Co. Ltd. (SCXK 2022–0011). Except for the normal group, the remaining rats were randomly divided into three groups (model group, acupuncture group, and sham acupuncture group) after modeling. After excluding depressed rats, 10 rats per group were included. The rats were acclimatized for 3 days in standard laboratory cages with ad libitum access to food and water. The housing conditions included a 12‐h light/dark cycle, a room temperature of 21°C ± 2°C, and a relative humidity of 50%–60%. The environment was maintained in a quiet, low‐stress setting to minimize external influences on the experimental outcomes. All experimental procedures were conducted following the ethical guidelines set by the Hainan Medical University Animal Ethics Committee (Approval HYLL‐2022‐019), ensuring humane treatment of the animals throughout the study.

The CUS model was used to induce anxiety‐like behaviors. Starting on day four post‐acclimatization, all groups except the normal group were exposed to 21 consecutive days of CUS, which included 12 randomly assigned stressors: water and food deprivation for 1–2 h, continuous 24‐h bright light or darkness, mild foot shocks (0.5 mA, 3 s), single housing isolation, cage tilting at a 45° angle for 1–2 h, cage mixing (placing 1–2 rats from two different cages together), forced swimming in 25°C water for 5 min, restraint in a ventilated tube for 1–2 h, tail clamping for 5 min, and intermittent exposure to an empty bottle to simulate unpredictable stress. These stressors were randomly distributed across the 21 days using the SPSS 22.0 statistical software, with 1–2 stressors applied daily to maintain unpredictability and avoid habituation. After the model was completed, rats were excluded if they ceased attempting to escape during foot shocks and met at least two of the following conditions: (1) a sucrose preference rate lower than 60% in the Sucrose Preference Test; (2) an immobility time in the 5‐min Forced Swim Test exceeding the control group mean by more than two standard deviations (SD); (3) movement time in the Open Field Test falling below two SDs from the control group mean.

### 3.2 Treatments

#### 3.2.1 Electroacupuncture Group

Acupuncture needles (0.25 mm × 25 mm and 0.25 mm × 13 mm) were obliquely inserted into the acupoints Baihui (GV20), Taichong (LV3), and vertically inserted into the acupoints Neiguan (PC6), and Shenmen (HT7). The insertion depth was approximately 5 mm at GV20 and LV3, and 2–3 mm at PC6 and HT7, consistent with standard acupuncture protocols. Treatments began daily at 9:00 a.m., with needle insertion followed by connection to a Han's Acupoint Nerve Stimulator (HANS‐200A). Continuous wave electrical stimulation was applied at a frequency of 2/15 Hz, with the intensity adjusted to elicit a mild, observable tremor in the limbs. Each EA session lasted 25 min and was administered once daily for 21 consecutive days. During treatment, rats were observed to ensure consistent needle placement and physiological responses.

#### 3.2.2 Normal Control ( **NC** ) and **CUS** Model Groups

In the normal control and CUS model groups, the animals were handled similarly to the EA group at 9:00 a.m. daily, with the same restraint protocol but without acupuncture treatment.

#### 3.2.3 Sham Acupuncture ( **SA** ) Group

In the sham acupuncture group, Blunt‐tipped needles were used to apply pressure to non‐acupoint locations 1 mm away from the actual acupoints to mimic the needling sensation experienced during acupuncture without piercing the skin, and treatment sessions lasted 25 min to match the EA group's duration. This setup ensured that any observed effects were specific to the EA treatment rather than handling or superficial needling alone.

### 3.3 Outcomes

#### 3.3.1 Body Weight Monitoring

The body weight of each rat in all groups was recorded on days 0, 7, 14, 21, 28, 35, and 42 of the experiment to monitor general health and assess the effects of the treatments.

#### 3.3.2 Sucrose Preference

After sucrose acclimation, rats were singly housed to assess their preference, with two pre‐weighed bottles provided: 1% sucrose solution and plain water for 24 h. Switch bottle positions midway to avoid side bias. Measure intake and calculate sucrose preference as (sucrose intake/total fluid intake) × 100%.

#### 3.3.3 Open Field Test (OFT)

The OFT was used to assess locomotor activity and anxiety‐like behavior. The test arena consisted of a 100 cm × 100 cm × 100 cm open field made of ABS material (provided by Hainan Medical University), with a black floor and white walls. An overhead infrared camera connected to the Smart v3.0 behavioral analysis system (Panlab, Spain) was used to track each rat's movements, with the arena divided into a central area (25%) and a peripheral area (75%). Each rat was tested for 5 min, and the arena was cleaned with 75% ethanol between tests to eliminate olfactory cues. The observation parameters included (1) total distance traveled: the total horizontal movement distance of the rat; (2) average speed: the ratio of the total distance traveled to the time spent moving; (3) time spent in the central zone: the amount of time the rat spent in the central zone, including both movement and stationary time.

#### 3.3.4 Elevated Plus Maze Test (EPM)

The EPM was utilized to evaluate anxiety‐like behavior induced by open spaces and heights. The maze, provided by Hainan Medical University, consisted of two open arms and two enclosed arms, elevated 50 cm above the floor. An infrared camera connected to the Smart v3.0 system tracked the rats' movements. Each rat was placed on the central platform and the following parameters were recorded. (1) percentage of open arm entries (OE%): the percentage of entries into any of the open arms, defined by the rat entering with all four paws. Calculated as OE% = OE/(OE + CE) × 100%, where OE is the number of open arm entries, and CE is the number of closed arm entries. (2) percentage of time spent in open arms (OT%): the cumulative time spent in the open arms, measured in seconds (s). Calculated as OT% = OT/(OT + CT) × 100%, where OT is the time spent in open arms, and CT is the time spent in closed arms. After each session, the apparatus was cleaned with 75% ethanol to prevent scent contamination.

#### 3.3.5 Real‐Time PCR for Gene Expression

Following behavioral tests, rats were fasted (but with free access to water) for 24 h before being sacrificed. The brain was rapidly extracted, rinsed in distilled water to remove residual blood, and placed on ice for amygdala and medial prefrontal cortex (mPFC) tissue dissection. Total RNA was extracted from these regions, and cDNA was synthesized for real‐time PCR using a kit from Servicebio (Wuhan, China) according to the manufacturer's instructions. Primers, synthesized by Giga (Shenzhen, China), were used to detect mRNA levels of IGF‐1 in the mPFC, and NR2B, GluR1, GluR2, IGF‐1 in the amygdala. The primer sequences were shown in Table S1.

#### 3.3.6 Western Blot Analysis

The dissected amygdala and mPFC tissues were homogenized in RIPA lysis buffer containing protease and phosphatase inhibitors, PMSF, and 2 mm grinding beads, using a tissue grinder (−20°C, 14000 rpm, 2 min). Protein concentrations were quantified by the BCA Protein Assay Kit (Beyotime Biotechnology, Shanghai, China). Proteins were separated by SDS‐PAGE and transferred to PVDF membranes, which were incubated with the following primary antibodies: IGF‐1 (Rabbit monoclonal, 1:1000), GluR1 (Rabbit monoclonal, 1:1000), GluR2 + 3 (Rabbit monoclonal, 1:10000) from Abcam, NR2B (Rabbit polyclonal, 1:2000) from Proteintech (Wuhan, China), and Anti‐beta actin (Rabbit, 1:5000) from Servicebio (Wuhan, China). Membranes were then incubated with secondary antibody, goat anti‐rabbit IgG (1:10000) from Servicebio (Wuhan, China). Bands were visualized, and relative protein levels were analyzed.

#### 3.3.7 Immunofluorescence Staining for NR2B Expression

Rat brain sections containing the amygdala were paraffin‐embedded and sectioned at 5 μm thickness using a microtome (Leica Microsystems, Germany). Sections were deparaffinized, rehydrated, and antigen retrieval was performed in EDTA buffer. After washing in PBS (pH 7.4), sections were blocked with 1% BSA and 10% donkey serum for 30 min. Slides were incubated overnight at 4°C with NR2B primary antibody (Rabbit polyclonal, 1:100, Proteintech) and subsequently with Cy3 conjugated goat anti‐rabbit IgG secondary antibody (1:500, Servicebio) at room temperature for 50 min in the dark. DAPI was used to counterstain nuclei, and images were captured using a fluorescence microscope. ImageJ software was used to quantify fluorescence intensity.

### 3.4 Statistical Analysis

SPSS 22.0 and GraphPad Prism 9.0 were used for data analysis and graphing. Baseline characteristics for categorical variables (e.g., gender) were compared between groups using the chi‐square test. The Shapiro–Wilk test was used to assess the normality of continuous variables. Normally distributed continuous data were expressed as mean ± standard deviation (mean ± sd), and paired samples *t*‐test was used for intra‐group comparison. Post hoc comparisons using Tukey's HSD test were conducted following a significant omnibus one‐way ANOVA to identify which specific groups differed at the same time point. The non‐normally distributed continuous data were expressed as median (P25, P75), and the difference was statistically significant by Wilcoxon signed‐rank test, and inter‐group comparisons among multiple independent groups were conducted using the Kruskal‐Wallis test. If the Kruskal‐Wallis test was significant, post‐hoc pairwise comparisons were performed using the Dunn‐Bonferroni test. The fMRI data were paired by DPABI 7.0 paired *t*‐test and GRF (voxel *p* < 0.001, cluster *p* < 0.05) calibrated, and the correlation analysis between the scale score and the fMRI data was analyzed by Pearson or Spearman correlation analysis, respectively, and *p <* 0.05 was statistically significant.

## 4 Results

### 4.1 Clinical Study

A total of 34 patients with GAD were included in the acupuncture treatment group; however, 1 case was excluded due to excessive head motion data. The wait‐list control group consisted of 30 patients, with 3 dropping out due to worsening symptoms and medication usage, and 2 cases excluded for excessive head motion. There were no significant statistical differences (*p >* 0.05) in baseline characteristics such as gender, age, GAD‐7 scores, HAMA scores, and BDI scores between the acupuncture group and the wait‐list group before treatment, as shown in Table S2. All medication details for both groups are shown in Table S3.

### 4.2 Comparison of HAMA Scores

**FIGURE 2:**
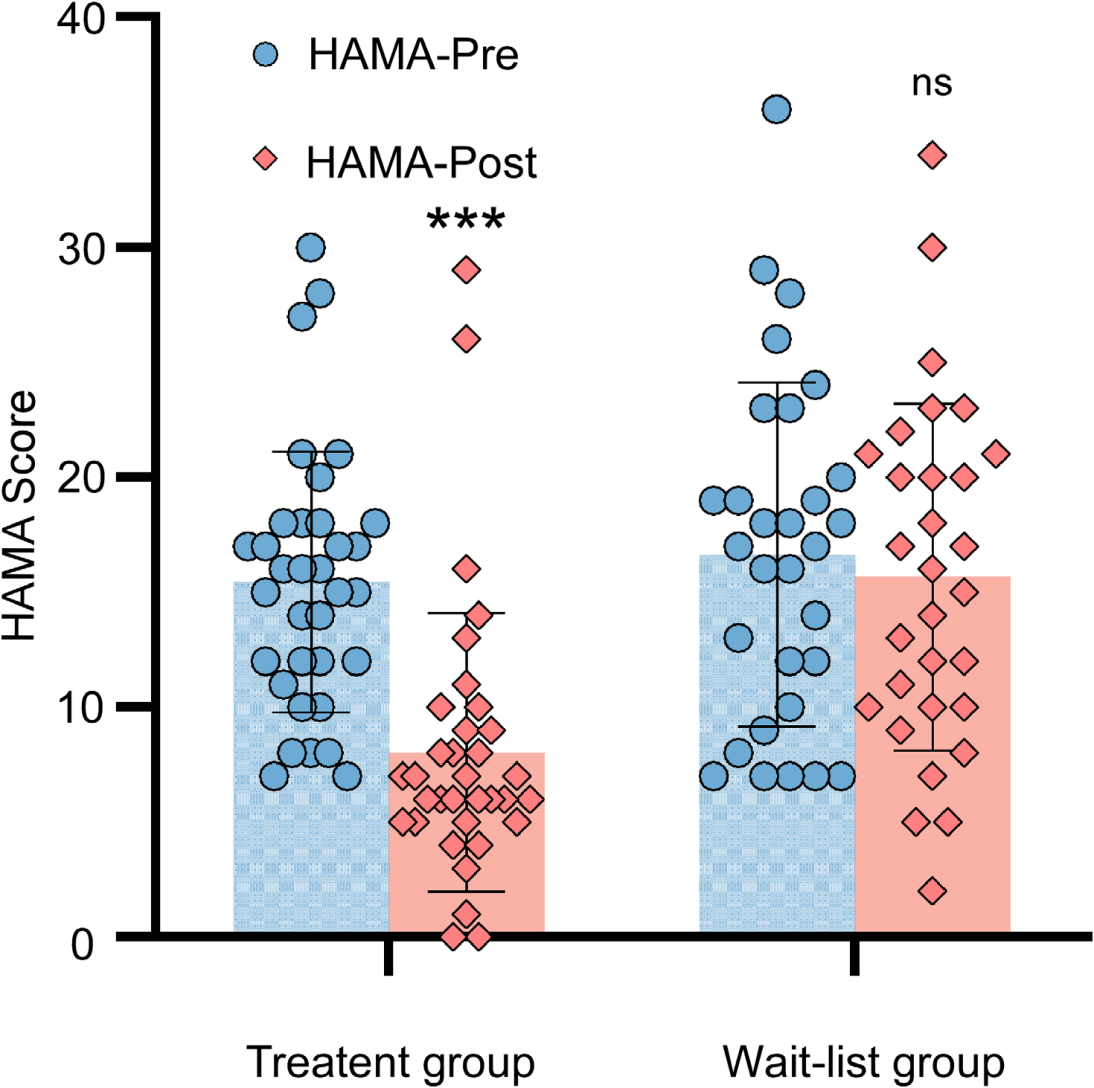
Comparison of pre‐ and post‐treatment HAMA scores in the acupuncture treatment group and the wait‐list group. Pre‐treatment: Before acupuncture; Post‐treatment: After acupuncture. Compared with pre‐treatment, ***: *P <* 0.001, ns: *P >* 0.05.

A comparison of HAMA scores before and after acupuncture treatment showed a significant decrease in scores, with a statistically significant difference (*p <* 0.001). In the wait‐list control group, the second assessment scores decreased compared to the first assessment, but no statistical significance was observed (*p >* 0.05), as shown in Figure 2.

### 4.3 Differences in ReHo Values in Resting‐State Brain Functional Imaging

**FIGURE 3:**
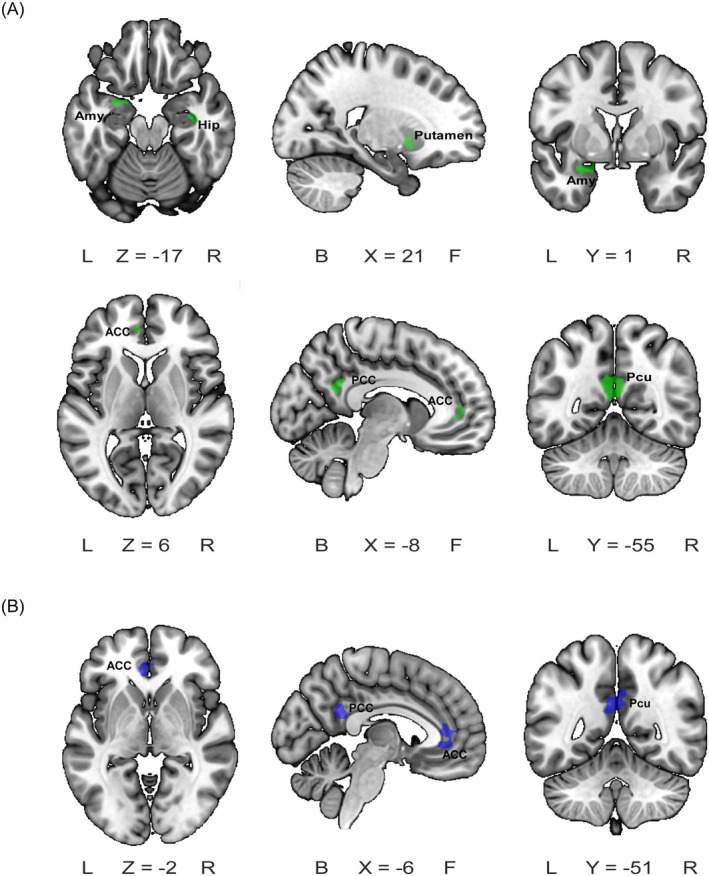
Differences in ReHo brain regions in resting‐state brain functional imaging. (A) Before and after acupuncture treatment; (B) Before and after in the wait‐list group. The blue regions represent brain areas where ReHo values decreased after treatment. ACC: Anterior Cingulate Cortex, Amy: Amygdala, Hip: Hippocampus, PCC: Posterior Cingulate Cortex, Putamen, Pcu: Precuneus. No significant increase in ReHo was observed in either the acupuncture group (pre‐ vs. post‐treatment) or the waitlist group (pre‐ vs. post‐observation).

In the acupuncture group for patients with GAD, ReHo values in key limbic system brain regions were significantly reduced after acupuncture treatment compared to pre‐treatment levels. The brain regions with reduced ReHo values primarily included the amygdala, hippocampus, posterior cingulate cortex, putamen, and precuneus (voxel *p* < 0.001, GRF correction, cluster *p* < 0.05), anterior cingulate cortex (voxel *p* < 0.005, GRF correction, cluster *p* < 0.05). Details are presented in Table S4 and Figure 3A.

Similarly, in the wait‐list group, ReHo values in key limbic system brain regions also decreased. The regions showing decreases primarily included the anterior cingulate cortex, posterior cingulate cortex, and precuneus (voxel *p* < 0.001, GRF correction, cluster *p* < 0.05). Details are presented in Table S5 and Figure 3B.

### 4.4 Correlation Between HAMA Score Change and ReHo Difference

**FIGURE 4:**
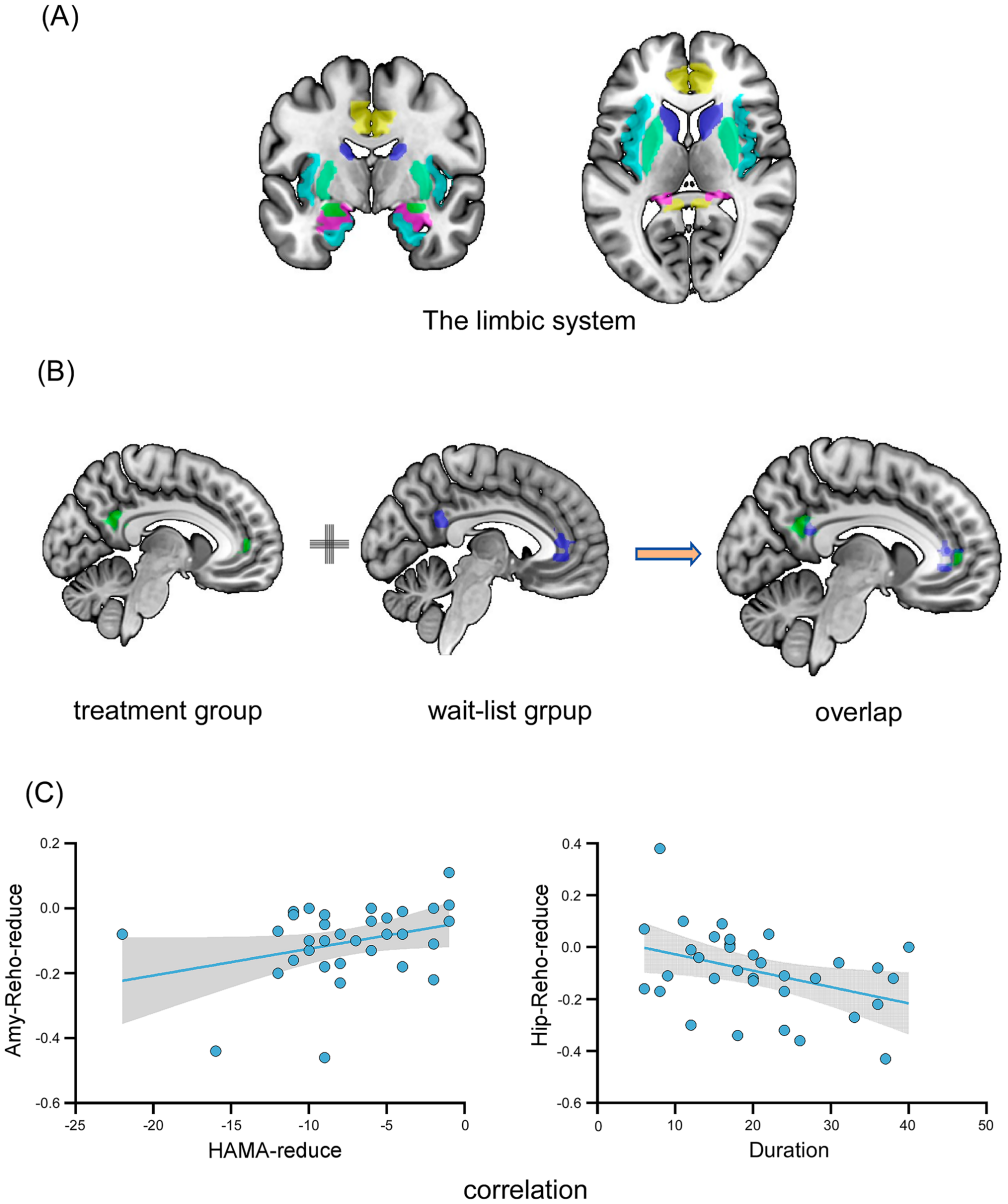
Overlapping brain regions and correlation analysis of ReHo value changes. (A) MASK image of limbic system brain regions; (B) Overlapping brain regions between the GAD acupuncture group and the wait‐list group; (C) Correlation analysis between the decrease in ReHo values in the amygdala and the difference in HAMA scores before and after treatment, and the correlation analysis between the decrease in ReHo values in the hippocampus and the duration of illness. Amy: Amygdala, Hip: Hippocampus.

In the acupuncture group, there was significant overlap with the wait‐list group in terms of decreased functional regions in the anterior cingulate cortex and posterior cingulate cortex. Nine voxels overlap in the anterior cingulate cortex, 17 voxels overlap in the posterior cingulate cortex. A correlation analysis was conducted between the reduction in ReHo values in brain regions that showed significant differences before and after acupuncture treatment in GAD patients and the difference in HAMA scores as well as the duration of illness. The results demonstrated a linear positive correlation between decreased ReHo values in the amygdala and the reduction in HAMA score (rs = 0.390, *p =* 0.023), and a linear negative correlation between decreased ReHo values in the hippocampus and the duration of illness (rs = −0.385, *p =* 0.025). See Figure 4.

### 4.5 Animal Experiments

**FIGURE 5:**
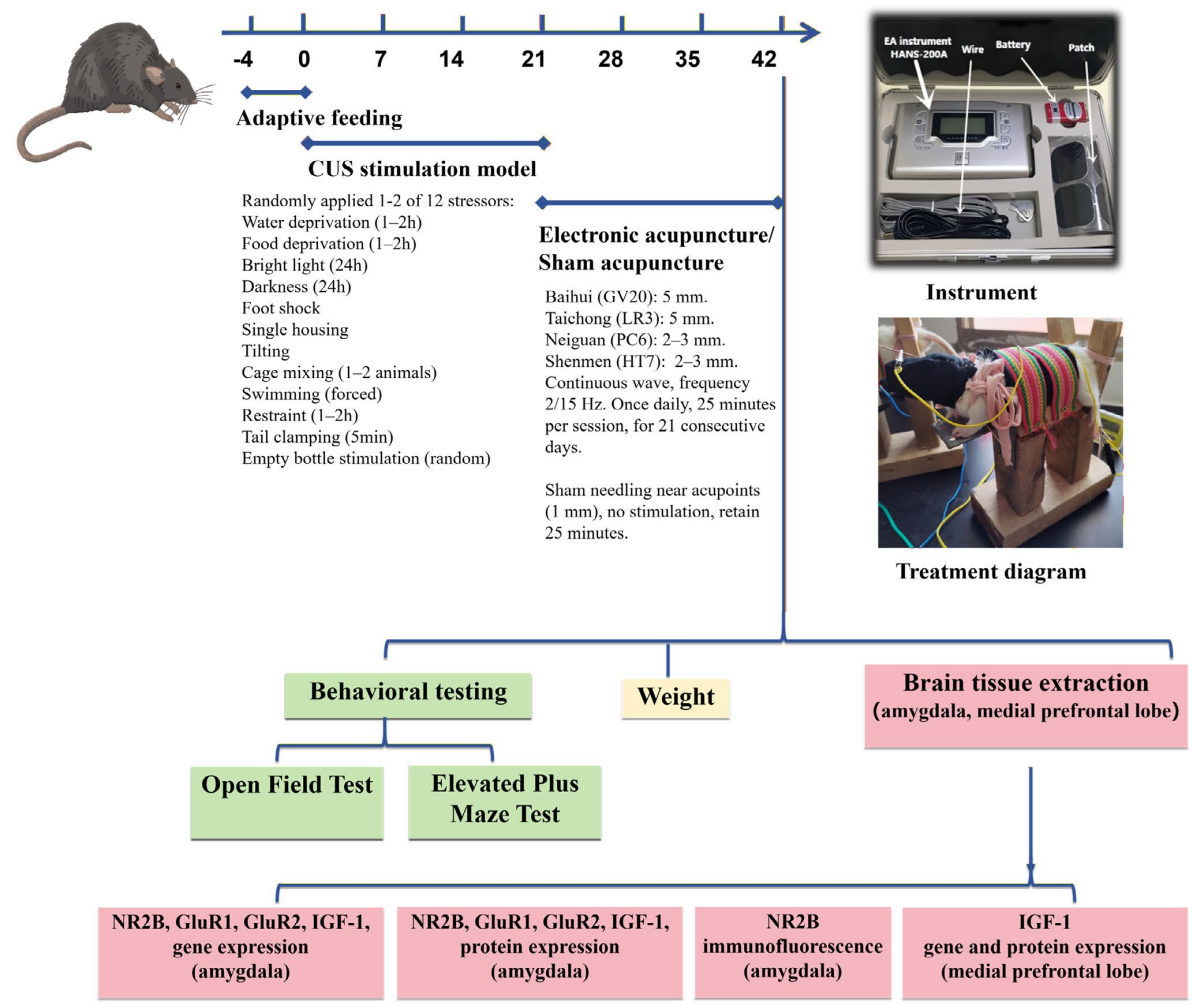
Flowchart of the animal experiments.

Based on the results of clinical studies, we further explored the central anxiolytic mechanisms of the EA on the medial prefrontal cortex‐amygdala axis in CUS model rats. The flowchart is shown in Figure 5.

### 4.6 Electroacupuncture Promoted Weight Gain in Rats

**FIGURE 6:**
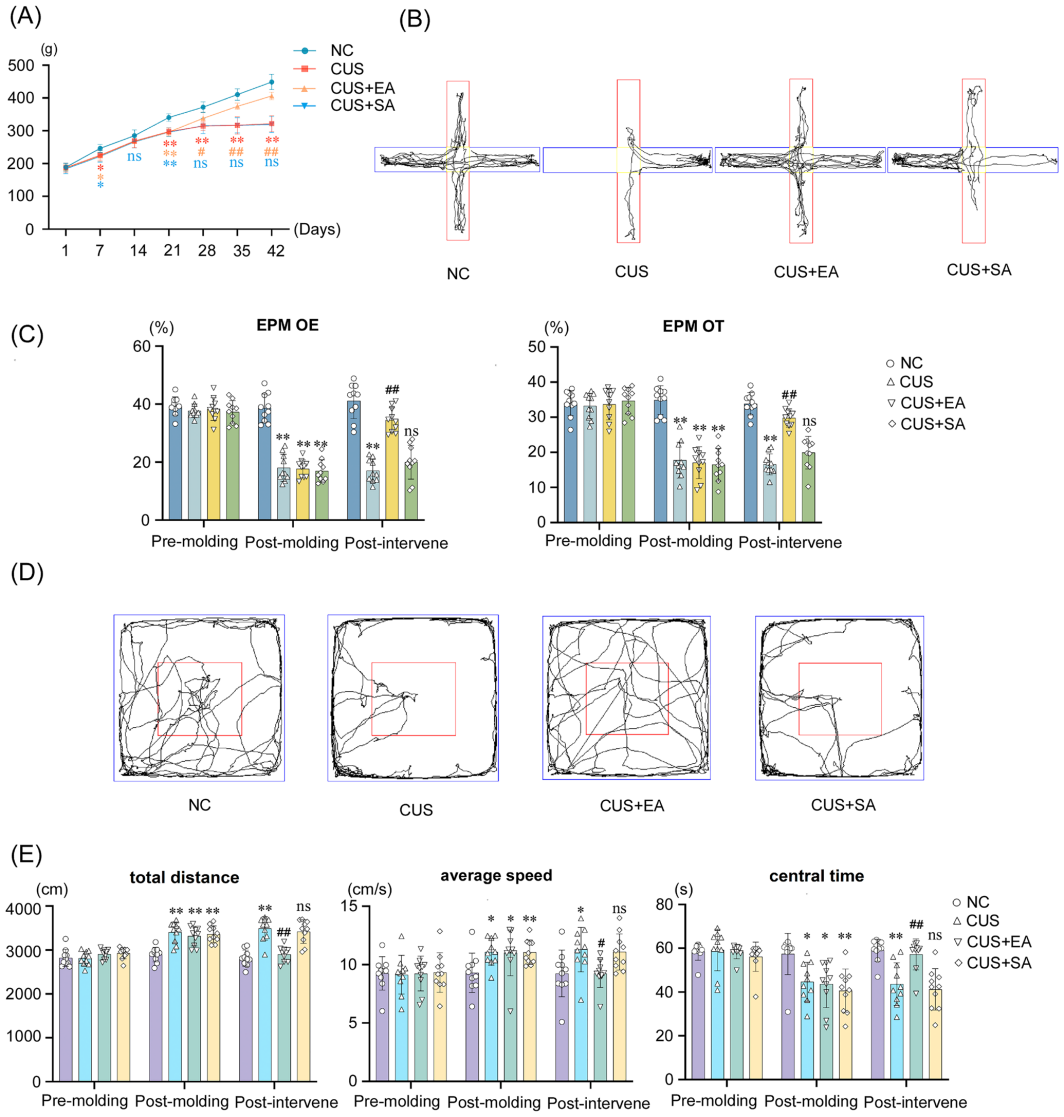
Weight and behavioral changes after treatment. (A) Weight; (B) Track plots of the rats in the elevated plus maze (EPM) experiment, the red area represents the open‐arm region, the blue area represents the closed‐arm region, and the yellow area is the central region; (C) Open arm entries (OE%) and Open arm time (OT%) in EPM; (D) Track plots of the rats in the Open field test (OFT) experiment, blue indicates the edge of the arena, and red indicates the central area; (E) Total distance in OFT, Average speed in OFT, Central time in OFT. NC: Normal control group; CUS: Model group; CUS + EA: Model + Electroacupuncture group; CUS + SA: Model + Sham acupuncture group. Compared to the NC group at the same time point: *p < 0.05, **p < 0.01. Compared to the CUS group at the same time point: ^#^ *p <* 0.05, ^##^ *p <* 0.01. ns indicates *p >* 0.05.

The weight of the CUS, EA, and SA groups significantly decreased compared to the NC group on days 7 and 21 of modeling (*p <* 0.05). Treatment intervention commenced following successful modeling on day 21. During the intervention period, the weight of the model group remained significantly lower than that of the NC group on days 28, 35, and 42 (*p <* 0.01). In comparison to the CUS group, the EA group exhibited a significant increase in weight (*p <* 0.05), whereas the SA group showed no significant difference (*p >* 0.05) (Table S6 and Figure 6A).

### 4.7 Sucrose Preference

The preference for sugar water in the normal group and the model group showed no significant statistical difference (*p* > 0.05), as shown in Table S7.

### 4.8 Elevated Plus Maze

After 21 days of modeling, the open arm time percentage (OT%) and open arm entries percentage (OE%) in the CUS group, EA group, and SA group were significantly lower compared to the NC group (*p <* 0.01), showing statistically significant differences. Following 3 weeks of intervention treatment, the CUS group continued to exhibit significantly reduced OT% and OE% compared to the NC group (*p <* 0.01), with statistically significant differences. Compared to the CUS group, the EA group showed a significant increase in OT% and OE% (*p <* 0.01), while the SA group demonstrated no significant differences in OT% or OE% (*p >* 0.05) (Figure 6B,C, Table S8).

### 4.9 Open Field Test

After 21 days of modeling, the CUS, EA, and SA groups showed significantly increased total travel distance and average speed compared to the NC group (*p <* 0.05), while the time spent in the central area was significantly reduced in all three groups (*p <* 0.05), indicating statistically significant differences. After 3 weeks of intervention treatment, the CUS group continued to exhibit significantly increased total travel distance and average speed (*p <* 0.05) and reduced central area residence time (*p <* 0.01) compared to the NC group. However, in comparison to the CUS group, the EA group demonstrated significantly reduced total travel distance and average speed (*p <* 0.05) and significantly increased central area residence time (*p <* 0.01), with statistically significant differences, while the SA group showed no significant differences in total travel distance, average speed, or central area residence time (*p >* 0.05) (Figure 6D,E, Table S8).

### 4.10 EA Modulates Neurotransmitter‐Related Gene Expression in the Amygdala and mPFC

**FIGURE 7:**
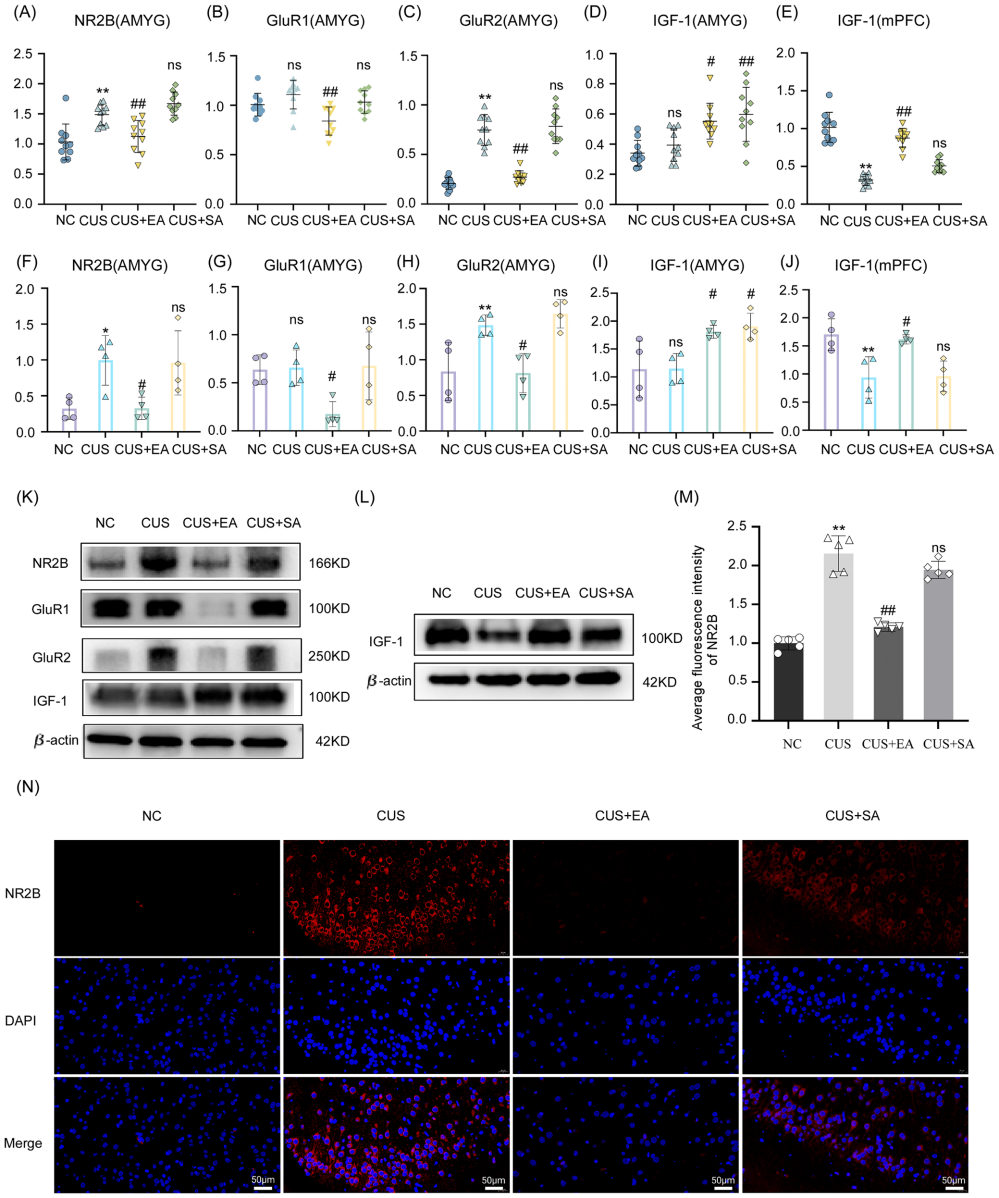
The regulatory effect of EA on neurotransmitter expression in the amygdala and medial prefrontal cortex of CUS Model Rats. (A–E) Relative mRNA expression levels of NR2B, GluR1, GluR2, IGF‐1 in the amygdala, and IGF‐1 in the mPFC across the NC, CUS, CUS + EA, and CUS + SA groups; (F–J) Quantitative analysis of protein expression levels of NR2B, GluR1, GluR2, IGF‐1 in the amygdala, and IGF‐1 in the mPFC; (K) Representative Western blot images of NR2B, GluR1, GluR2, and IGF‐1 in the amygdala and IGF‐1 in the mPFC; (L) Western blot image showing IGF‐1 protein expression in the amygdala; (M) Quantification of average fluorescence intensity of NR2B in the amygdala across the NC, CUS, CUS + EA, and CUS + SA groups; (N) Immunofluorescence images showing NR2B expression in the amygdala, stained with NR2B (red) and DAPI (blue). MPFC: Medial prefrontal cortex; NC: Normal control group; CUS: Model group; CUS + EA: Model + Electroacupuncture group; CUS + SA: Model + Sham acupuncture group. Compared to the NC group at the same time point: *p < 0.05, **p < 0.01. Compared to the CUS group at the same time point: ^#^ *p <* 0.05, ^##^ *p <* 0.01. ns indicates *p >* 0.05.

Compared to the NC group, the CUS group exhibited significantly reduced IGF‐1 mRNA expression in the medial prefrontal cortex (*p <* 0.01), while NR2B and GluR2 mRNA expression in the amygdala were significantly increased (*p <* 0.01). No significant changes were observed in GluR1 or IGF‐1 mRNA expression in the amygdala (*p >* 0.05). Compared to the CUS group, the EA group showed significantly increased IGF‐1 mRNA expression in the medial prefrontal cortex (*p <* 0.01) and significantly elevated IGF‐1 (*p <* 0.05) mRNA expression in the amygdala. Additionally, NR2B, GluR2, and GluR1 mRNA expression in the amygdala were significantly reduced (*p <* 0.01). In the SA group, no significant differences were observed in NR2B, GluR1 or GluR2 mRNA expression in the amygdala or IGF‐1 mRNA expression in the medial prefrontal cortex (*p >* 0.05), although IGF‐1 mRNA expression in the amygdala was significantly increased (*p <* 0.01) (Figure 7A–E).

### 4.11 EA Modulates Neurotransmitter‐Related Protein Expression in the Amygdala and mPFC

Compared to the NC group, the CUS group exhibited significantly reduced IGF‐1 protein expression in the medial prefrontal cortex (*p <* 0.01). Additionally, NR2B (*p <* 0.05) and GluR2 (*p <* 0.01) protein expression in the amygdala were significantly increased, while no significant changes were observed in GluR1 or IGF‐1 protein expression in the amygdala (*p >* 0.05). Compared to the CUS group, the EA group showed significantly increased IGF‐1 protein expression in the medial prefrontal cortex (*p <* 0.01) and significantly elevated IGF‐1 protein expression in the amygdala (*p <* 0.05), while NR2B (*p <* 0.05), GluR1 (*p <* 0.05), GluR2 (*p <* 0.01) protein expression in the amygdala were significantly reduced. In the SA group, no significant differences were observed in the expression of NR2B, GluR1, GluR2 proteins in the amygdala or IGF‐1 protein expression in the medial prefrontal cortex (*p >* 0.05), while the expression of IGF‐1 in the amygdala also increased (*p <* 0.05) (Figure 7F–L).

### 4.12 EA Reduces Average Fluorescence Intensity of NR2B in the Amygdala

Observed under a fluorescence microscope at 400 **×** magnification, the average fluorescence intensity of NR2B in the amygdala varied among the groups. Compared to the NC group, the CUS group exhibited a significant increase in the average fluorescence intensity of NR2B in the amygdala (*p <* 0.01), showing statistical significance. In contrast, the EA group showed a significant reduction in NR2B fluorescence intensity compared to the CUS group (*p <* 0.01), with statistical significance. However, no significant differences were observed in NR2B fluorescence intensity in the SA group compared to the CUS group (*p >* 0.05) (Figure 7M,N).

## 5 Discussion

This study provided comprehensive evidence from both clinical and animal research supporting the anxiolytic effects of acupuncture in GAD. The study highlighted the role of acupuncture in rebalancing the medial prefrontal cortex–amygdala axis, a key pathway implicated in GAD and widely discussed in anxiety‐related neurocircuitry. Through its effects on neurotransmitter systems, this treatment not only alleviates anxiety symptoms but also sheds light on the mechanisms of neural regulation.

Clinically, we observed that both the acupuncture group and the wait‐list group exhibited decreased functional activity in the ACC and PCC during the second scan. This reduction in activity did not show a clear correlation with changes in anxiety levels, possibly due to the fixed scan‐treatment/wait‐scan sequence. The ACC plays a crucial role in attention allocation and control, being activated in tasks requiring selective attention, such as focusing on specific sounds in a noisy environment. Similarly, the PCC is involved in directing attention, especially in spatial and endogenous attention tasks, working with other brain regions to guide attention orientation. During the first scan, participants were likely distracted by the new environment. In the second scan, decreased activation in the ACC and PCC may partly reflect habituation to the scanning environment. The precuneus helps focus on specific targets, filtering out irrelevant information [25].

It is noteworthy that GAD is associated with alterations in threat processing that can be described by two complementary neural pathways: a quick but less precise response via direct thalamic input to the amygdala's basolateral nucleus and onward to its central nucleus, and a slower but more refined response via the thalamus and higher cortical structures for processing before engaging the amygdala [26]. This supports increased amygdala and entorhinal cortex activation reported in GAD and in several anxiety‐related samples compared to healthy individuals [27]. Following an eight‐week mindfulness‐based intervention, amygdala activation decreased with enhanced connectivity to prefrontal areas [28]. Moreover, hippocampal neuron excitability is associated with anxiety, as seen in animal studies where anxiety‐prone mice showed significant activation in the hippocampus [29]. Changes in hippocampal plasticity might disrupt its function in GAD, as some anxiolytics modulate hippocampal excitability and plasticity [30]. Early‐onset mental disorders also relate to abnormal hippocampal development, which plays a key role in GAD [31]. The amygdala and hippocampus closely interact, regulating emotions, yet this connectivity may be impaired in GAD [32]. The putamen also participates in emotional regulation circuits involving key brain regions and neurotransmitter systems, affecting emotional expression and related habitual behaviors [33].

The animal experiments complemented these clinical findings by elucidating molecular changes in the mPFC‐amygdala axis. Chronic stress‐induced anxiety behaviors were accompanied by heightened expression of NR2B, markers of glutamatergic overactivation, as well as reduced expression of IGF‐1, reflecting impaired inhibitory signaling and neurotrophic support. Electroacupuncture reversed these molecular changes, suppressing excitatory neurotransmission and enhancing inhibitory signaling. These effects are consistent with prior research showing acupuncture's ability to modulate glutamate systems in stress models.

These molecular findings from animal experiments suggest a convergent circuit‐level mechanism for the induced reduction in amygdala ReHo observed in clinical functional imaging. The suppression of NR2B expression and enhancement of inhibitory signaling in the mPFC‐amygdala circuit likely underlie the restoration of excitatory/inhibitory balance, which manifests as decreased ReHo values indicating normalized neural synchronization. Similarly, EA's modulation of glutamatergic systems may contribute to reduced amygdala hyperexcitability and altered functional connectivity patterns. These complementary findings across species suggest convergent molecular and network‐level mechanisms through which EA exerts its anxiolytic effects. These associations require further validation through multimodal neuroimaging combined with molecular assays in human studies.

This study aligned with previous research in emphasizing the critical roles of glutamatergic systems and IGF‐1 in anxiety regulation, demonstrating that chronic stress‐induced glutamatergic overactivation, such as NR2B, contributed to anxiety‐like behaviors [7, 34, 35]. Recent studies have further elucidated these mechanisms. Yin et al. demonstrated that EA attenuates chronic stress‐induced anxiety through suppressing NOX2‐derived oxidative stress in the ventral hippocampus [36], while Li et al. showed EA reduces microglial hyperactivity and synaptic phagocytosis in the mPFC to alleviate pain‐comorbid anxiety [37]. It also corroborated findings that acupuncture can normalize neurotransmitter imbalances by suppressing excitatory markers and enhancing inhibitory signaling, thereby alleviating anxiety symptoms [38], as evidenced by Yang et al.'s report of EA modulating LCN2‐mediated neuroinflammation in the prefrontal cortex to improve PTSD‐related anxiety behaviors [39]. Moreover, the increase in IGF‐1 expression in the mPFC observed in this study supports earlier reports of IGF‐1's neuroprotective and anxiolytic effects, including its role in mitigating stress vulnerability and restoring synaptic plasticity [40, 41], complementing Fang et al.'s findings that EA regulates astrocyte inhibition and parvalbumin interneuron activation in pain‐anxiety comorbidity [42]. Our study focused on the mPFC‐amygdala axis, a key circuit underlying anxiety. Unlike prior research that often isolated individual brain regions, this study provided a more integrated view of anxiety pathophysiology. Furthermore, the integration of clinical and preclinical findings allowed direct comparisons between human and animal models, bridging the gap between experimental and clinical studies. The identification of specific molecular targets, such as NR2B, GluR2, and IGF‐1, offers deeper insights into how acupuncture modulates these pathways, expanding on prior studies that largely focused on broad neurotransmitter effects [38, 41].

While this study provided robust evidence for the anxiolytic mechanisms of acupuncture, several limitations should be addressed. First, the clinical sample size was relatively small, and larger trials are needed to confirm these findings. Second, the animal model, while informative, may not fully recapitulate the complexity of human anxiety disorders. In addition, cross‐species inference is constrained by differences in medial prefrontal anatomy and functional parcellation between rodents and humans, and our interpretation emphasizes conserved circuit functions rather than strict anatomical correspondence. Therefore, the integration of human imaging and rat molecular data is intended to capture functional analogy at the circuit level, focusing on convergent evidence for prefrontal regulation of amygdala‐related processing and shared molecular signatures of excitatory‐inhibitory balance, rather than direct region‐to‐region equivalence [43, 44]. For animal experiments, standardized electroacupuncture with fixed parameters was implemented due to animals' inability to provide needling sensation feedback and manual manipulation's inconsistent stimulation intensity. While this methodological constraint may influence data interpretation, it ensures acupoint‐specific therapeutic effects remain measurable. Future studies should explore the molecular mechanisms underlying the effects of acupuncture in greater detail, including its impact on neuroinflammation and synaptic plasticity. Additionally, investigating the long‐term efficacy and safety of acupuncture in diverse populations would further strengthen its clinical utility.

## 6 Conclusion

This study demonstrated that acupuncture effectively alleviates anxiety by restoring behavioral, physiological, and molecular homeostasis in GAD patients and CUS model rats. Clinically, acupuncture significantly reduced anxiety symptoms and altered activity in brain regions such as the amygdala and hippocampus, contributing to improved emotional regulation. By targeting the mPFC‐amygdala axis, acupuncture rebalances excitatory and inhibitory signaling, reduces hyperactivation in anxiety‐related regions, and enhances emotion regulation. These findings provide a foundation for the clinical application of acupuncture in managing GAD. The extent to which these mechanisms generalize to other anxiety disorders requires direct testing in disorder‐specific cohorts.

## Author Contributions

Z.S. and W.S. contributed to the study design and drafting of the manuscript. Y.W., B.R., X.K., and Q.W. were responsible for data collection and analysis. L.H. and J.L. participated in the interpretation of the data and manuscript revisions. L.K., Y.W., and H.L. provided technical support and guidance throughout the study. J.L. and W.S. supervised the research project, providing critical insights and final approval of the manuscript. All authors read and approved the final manuscript.

## Funding

This work was supported by National Natural Science Foundation of China (81560797, 82260966); Hainan Provincial Natural Science Foundation (821RC567, 823RC492); and Hainan Provincial Health Industry Research (22A200035); Academic Enhancement Support Program of Hainan Medical University (XSTS2026199).

## Ethics Statement

Clinical trial number: MR‐46‐23‐043956 (www.yxyj.org.cn/www.medicalresearch.org.cn). Scientific title: Study on the Effect of Acupuncture on the Response Characteristics of Brain Functional Connection Networks in Patients with Generalized Anxiety. Ethics approval and consent to participate for this study were obtained from the Medical Ethics Committee of the First Affiliated Hospital of Hainan Medical University (Approval No. 2023‐KYL‐068). All participants provided informed consent prior to their involvement in the study. The animal experiments were conducted in accordance with the guidelines set by the Ethics Committee for Laboratory Animals at Hainan Medical University, under approval number HYLL‐2022‐019, ensuring compliance with ethical standards for the care and use of animals in research.

## Consent for publication

Not applicable.

## Conflicts of Interest

The authors declare no conflicts of interest.

## Supporting information


**Table S1:** Primer sequences.
**Table S2:** Baseline characteristics of the treatment and wait‐list groups.
**Table S3:** Comparison of drug use between the treatment group and wait‐list group.
**Table S4:** Differences in ReHo Values before and after acupuncture treatment.
**Table S5:** Differences in ReHo Values before and after wait‐list treatment.
**Table S6:** Effects of acupuncture on body weight changes in rats.
**Table S7:** Comparison of sucrose preference index between normal and Model groups at 24 h.
**Table S8:** Comparison of EPM and OFT behaviors across different groups.

## Data Availability

The data that support the findings of this study are available from the corresponding author upon reasonable request.
