# Supplementary material for: Acupuncture Modulates Neurotransmitter‐Related Molecules in the Amygdala to Ameliorate Generalized Anxiety Disorder
Source: CNS Neurosci Ther. 2026 Apr 20;32(4):e70847. doi: 10.1002/cns.70847 (PMC13093602; doi:10.1002/cns.70847)
Supplement: Supplementary file 1 — Table S1: Primer sequences. Table S2: Baseline characteristics of the treatment and wait‐list groups. Table S3: Comparison of drug use between the treatment group and wait‐list group. Table S4: Differences in ReHo Values before and after acupuncture treatment. Table S5: Differences in ReHo Values before and after wait‐list treatment. Table S6: Effects of acupuncture on body weight changes in rats. Table S7: Comparison of sucrose preference index between normal and Model groups at 24 h. Table S8: Comparison of EPM and OFT behaviors across different groups. [file CNS-32-e70847-s001.docx]

**Supplementary material**

**Table S1. Primer sequences**

| Gene | **Sequences** | |
| --- | --- | --- |
| NR2B | F | 5'-TCCTCCTGAGTCGGAGTGTG-3' |
|  | R | 5'-AAAGCGTCCCCTTCCGGTTC-3' |
| GluR1 | F | 5'-GGTTGTGGGTGCCAATTTCC-3' |
|  | R | 5'-GAACTGGGAACAGAAACGGT-3' |
| GluR2 | F | 5'-CGAGCAGAGGAAGCCTTGTG-3' |
|  | R | 5'-CCGAGTCCTTGGCTCCACAT-3' |
| IGF-1 | F | 5'-AGAGCCTGCGCAATCGAAAT-3' |
|  | R | 5'-TCGATAGGGGCTGGGACTTC-3' |
| ACTB | F | 5'-CTGACTGAGCGTGGCTATT-3' |
|  | R | 5'-AGGGAAGAAGAGGAAGCAG-3' |

**Table S2. Baseline Characteristics of the treatment and wait-list groups**

| Group | Gender  (male/female) | Age (year) | Duration (month) | BDI | GAD-7 | HAMA |
| --- | --- | --- | --- | --- | --- | --- |
| Treatment | 11/23 | 39.41±16.03 | 20.52±9.72 | 7.85±4.17 | 12.94±2.41 | 15.44±5.67 |
| Wait-list | 11/19 | 39.70±13.56 | 20.33±10.30 | 7.60±4.06 | 13.10±2.13 | 16.63±7.47 |

**Note:** Continuous variables are presented as mean ± SD and analyzed via independent t-tests. Gender is presented as counts and analyzed via Chi-square test. BDI: Beck Depression Inventory; GAD-7: Generalized Anxiety Disorder-7; HAMA: Hamilton Anxiety Rating Scale. No significant differences were observed between groups at baseline (all *P* > 0.05).

**Table S3.** Comparison of drug use between the treatment group and wait-list group

| Medication Class / Specific Drug Name | Treatment Group (n=34) | Wait-list Group (n=30) | Total (N=64) | *P* value |
| --- | --- | --- | --- | --- |
| Sedative-Hypnotics | 4 (11.8%) | 2 (6.7%) | 6 (9.4%) |  |
| Alprazolam | 2 | 0 | 2 |  |
| Estazolam | 1 | 0 | 1 |  |
| Lorazepam | 1 | 1 | 2 |  |
| Eszopiclone | 0 | 1 | 1 |  |
| Antidepressants & Anxiolytics | 2 (5.9%) | 3 (10.0%) | 5 (7.8%) |  |
| Flupentixol and Melitracen Tablets | 1 | 1 | 2 |  |
| Paroxetine | 1 | 2 | 3 |  |
| Total Medicated Patients | 6 (17.6%) | 5 (16.7%) | 11 (17.2%) | 0.963 |

**Note:** To address potential confounding factors related to concurrent medication, we compared the proportion of patients continuing their original drug regimens between the two groups. In the acupuncture group, 6 patients (17.6%) were taking sedative/hypnotic or antidepressant medications, compared to 5 patients (16.7%) in the wait-list control group. *P* value was calculated only for the overall proportion of medicated patients. Fisher's exact test showed no statistically significant difference in the proportion of medicated patients between the two groups (*P* = 0.963).

**Table S4.** **Differences in ReHo Values Before and After Acupuncture Treatment**

| Condition | Brain Region | Brodmann Area (BA) | Voxel Count | T-Value | MNI Coordinates (x, y, z) | *P* value | FWHM  X/Y/Z |
| --- | --- | --- | --- | --- | --- | --- | --- |
| **Post-acupuncture < Pre-acupuncture** | **Left Amygdala** | **BA34** | **17** | **5.19** | **-27, 0, -18** | *<0.0001* | 5.78mm/7.03mm/5.77mm |
|  | **Right Hippocampus** | **BA36** | **19** | **3.57** | **34, -15,-15** | *<0.001* |  |
|  | **Anterior Cingulate Cortex** | **BA10** | **19** | **3.24** | **-9, 45, 6** | *P=0.0025* |  |
|  | **Posterior Cingulate Cortex** | **BA23** | **61** | **4.37** | **-3, -45, 21** | *<0.0001* |  |
|  | **Putamen** | **BA48** | **50** | **3.95** | **30, -9, -6** | *<0.001* |  |
|  | **Precuneus** | **BA23** | **45** | **4.37** | **-6, -55, 24** | *<0.0001* |  |
| **Post-acupuncture > Pre-acupuncture** | None | None | None | None | None |  |  |

**Note:** The threshold was set at a voxel-level height threshold of *P* < 0.001(uncorrected) for initial cluster formation, with a cluster-level significance threshold of *P* < 0.05(GRF-corrected) to determine statistically significant clusters. Peak coordinates (MNI space) and cluster size (voxel count) are reported for each significant brain region. Significant findings were observed in the Anterior Cingulate Cortex (ACC) during acupuncture treatment under the statistical threshold of voxel *P* < 0.005 with Gaussian Random Field (GRF) correction (cluster-level *P* < 0.05).

**Table S5.** Differences in ReHo Values Before and After Wait-List Treatment

| Condition | Brain Region | Brodmann Area (BA) | Voxel Count | T-Value | MNI Coordinates (x, y, z) | *P* value | FWHM  X/Y/Z |
| --- | --- | --- | --- | --- | --- | --- | --- |
| Post-treatment < Pre-treatment | Anterior Cingulate Cortex | BA30 | 48 | 4.45 | -2, 37, -6 | <0.001 | 6.59mm/7.41mm/5.57mm |
|  | Posterior Cingulate Cortex | BA23 | 51 | 4.81 | 0, -54, 27 | <0.001 |  |
|  | Precuneus | BA23 | 25 | 4.63 | 2, -49, 33 | <0.0001 |  |
| Post-treatment  > Pre-treatment | None | None | None | None | None |  |  |

**Note:** The threshold was set at a voxel-level height threshold of *P* < 0.001 (uncorrected) for initial cluster formation, with a cluster-level significance threshold of *P* < 0.05 (GRF-corrected) to determine statistically significant clusters. Peak coordinates (MNI space) and cluster size (voxel count) are reported for each significant brain region.

**Table S6.** Effects of acupuncture on body weight changes in rats

| Group | Day 0 (g) | Day 7 (g) | Day 14(g) | Day21 (g) | Day28 (g) | Day35  (g) | Day42 (g) |
| --- | --- | --- | --- | --- | --- | --- | --- |
| **NC** | 189.37 ± 12.15 | 246.39 ± 12.08 | 285.34 ± 17.52 | 340.55±  11.76 | 372.15±  15.94 | 413.95(392.93, 428.25) | 448.85 ± 23.36 |
| **CES** | 187.78 ± 13.74 | 225.76 ± 14.99* | 269.11 ± 20.61 | 297.78±  10.14** | 314.47±  12.63** | 316.95(291.18, 339.05)** | 321.96 ± 24.12** |
| **CES+EA** | 186.59 ± 12.08 | 223.74 ± 17.17* | 267.75 ± 19.02 | 296.27±  12.49** | 338.14±  19.34^#^ | 373.25(369.58, 381.18)^#^ | 406.49 ± 11.30^##^ |
| **CES+SA** | 184.55 ± 14.87 | 222.44 ± 17.79* | 266.50 ± 18.36 | 295.97±  13.20** | 315.09±  23.28^ns^ | 323.95(294.48, 335.90)^ns^ | 319.40 ± 24.76^ns^ |
| ***F/K*** | 0.23 | 5.19 | 2.185 | 33.73 | 22.14 | 32.28 | 88.13 |
| *P* value | 0.8717 | 0.0044 | 0.1067 | ＜0.0001 | ＜0.0001 | ＜0.0001 | ＜0.0001 |

**Note:** Data are presented as mean ± SD or Median (Q1 Q3). The sample size for each group was 10 (n = 10). Statistical comparisons among groups at each time point were assessed by one‑way ANOVA with Tukey's post-hoc test for multiple comparisons. On Day 35, the Kruskal-Wallis test with Dunn's post-hoc test was used due to a deviation from normality. The corresponding F values (from ANOVA) or K values (from Kruskal-Wallis test) and exact *P* values are provided in the table. *P*<0.05 was considered statistically significant. **Compared to the NC group at the same time point:** **P* < 0.05, ***P* < 0.01. Compared to the CES group at the same time point: ^#^*P* < 0.05, ^##^*P* < 0.01. ns indicates *P* > 0.05.

**Table S7.** Comparison of sucrose preference index between Normal and Model groups at 24h

| Group | n | Sucrose Preference  (Mean ± SD) | t | *P* value |
| --- | --- | --- | --- | --- |
| **Normal** | 10 | 0.699 ± 0.140 | 1.012 | 0.325 |
| **Model** | 10 | 0.624 ± 0.188 |  |  |

**Note:** Data are presented as Mean ± SD. The independent samples t-test was used for statistical analysis. *P* > 0.05 indicates no significant difference between the two groups.

**Table S8.** Comparison of EPM and OFT behaviors across different groups

|  | Time | NC | CES | CES+EA | CES+SA | F | *P* value |
| --- | --- | --- | --- | --- | --- | --- | --- |
| **EPM** |  | | | | | | |
| Open Arm Entries (%) | Pre-modeling | 39.38±3.14 | 37.73±2.32 | 38.30±3.93 | 37.35±3.89 | 0.68 | 0.5688 |
|  | Post-modeling | 38.66±4.88 | 18.08±4.67** | 17.71± 3.07** | 16.98± 3.83** | 63.85 | <0.0001 |
|  | Final-treatment | 41.18±6.09 | 17.06±3.93** | 34.54± 4.23^##^ | 20.00± 5.85^ns^ | 50.97 | <0.0001 |
| Open Arm Time (%) | Pre-modeling | 34.05±3.58 | 33.23±3.62 | 33.70±4.43 | 34.68±3.76 | 0.25 | 0.8608 |
|  | Post-modeling | 34.90±4.10 | 17.77±5.08** | 17.06± 4.54** | 16.51± 4.65** | 37.41 | <0.0001 |
|  | Final-treatment | 33.84±3.19 | 16.59±2.99** | 29.80± 2.60^##^ | 19.92± 4.65^ns^ | 55.5 | <0.0001 |
| **OFT** |  | | | | | | |
| Total Distance (cm) | Pre-modeling | 2822.09±192.26 | 2813.91±155.79 | 2906.21±  115.74 | 2927.94±  126.53 | 1.48 | 0.2354 |
|  | Post-modeling | 2897.09±165.90 | 3404.60±223.11** | 3318.67± 223.20** | 3362.22± 190.41** | 13.19 | <0.0001 |
|  | Final-treatment | 2817.37±182.60 | 3491.63±265.66** | 2901.68± 169.80^##^ | 3419.05± 241.75^ns^ | 23.48 | <0.0001 |
| Average speed (cm/s) | Pre-modeling | 9.26±1.42 | 9.14±1.66 | 9.23±1.48 | 9.33±1.69 | 0.03 | 0.9941 |
|  | Post-modeling | 9.31±1.67 | 11.08±1.16* | 11.00±1.95* | 11.06±1.04** | 3.36 | 0.0291 |
|  | Final-treatment | 9.25±1.99 | 11.29±1.90* | 9.25±1.20^#^ | 11.09±1.55^ns^ | 4.42 | 0.0096 |
| Central Time (s) | Pre-modeling | 58.47±3.92 | 59.25±9.47 | 58.41±3.17 | 56.15±6.65 | 0.45 | 0.7200 |
|  | Post-modeling | 57.46±9.40 | 44.71± 8.88* | 43.67±10.7* | 40.64± 9.90** | 5.80 | 0.0024 |
|  | Final-treatment | 59.10±5.01 | 43.67±9.74** | 57.27±7.07 ^##^ | 41.22± 9.48^ns^ | 12.96 | <0.0001 |

**Note:** Data are presented as mean ± SD (n = 10). F values and *P* values were derived from one-way ANOVA comparing the four groups at each time point, with post-hoc correction using Tukey's method. **Compared to the NC group at the same time point:** **P* < 0.05, ***P* < 0.01. Compared to the CES group at the same time point: ^#^*P* < 0.05, ^##^*P* < 0.01. ns indicates *P* > 0.05.
